# Correction: Efficacy of surface-functionalized Mg_1−*x*_Co_*x*_Fe_2_O_4_ (0 ≤ *x* ≤ 1; Δ*x* = 0.1) for hyperthermia and *in vivo* MR imaging as a contrast agent

**DOI:** 10.1039/d2ra90032g

**Published:** 2022-04-13

**Authors:** M. Aminul Islam, M. Razibul Hasan, Md. Mahbubul Haque, Rimi Rashid, Ishtiaque M. Syed, S. Manjura Hoque

**Affiliations:** Materials Science Division, Atomic Energy Centre Dhaka, Bangladesh Atomic Energy Commission 1000 Dhaka Bangladesh manjura_hoque@yahoo.com; Magura Govt. Mahila College Magura Bangladesh; Department of Physics, University of Dhaka Bangladesh

## Abstract

Correction for ‘Efficacy of surface-functionalized Mg_1−*x*_Co_*x*_Fe_2_O_4_ (0 ≤ *x* ≤ 1; Δ*x* = 0.1) for hyperthermia and *in vivo* MR imaging as a contrast agent’ by M. Aminul Islam *et al.*, *RSC Adv.*, 2022, **12**, 7835–7849, DOI: 10.1039/D2RA00768A.

The authors regret that the name of one of the authors (Md. Mahbubul Haque) was shown incorrectly in the original article. The corrected author list is as shown here.

The Royal Society of Chemistry apologises for these errors and any consequent inconvenience to authors and readers.

## Supplementary Material

